# Effect of Aspect Ratio on the Permittivity of Graphite Fiber in Microwave Heating

**DOI:** 10.3390/ma11010169

**Published:** 2018-01-22

**Authors:** Jun Fukushima, Shuntaro Tsubaki, Tomoki Matsuzawa, Keiichiro Kashimura, Tomohiko Mitani, Tomoaki Namioka, Satoshi Fujii, Naoki Shinohara, Hirotsugu Takizawa, Yuji Wada

**Affiliations:** 1 Department of Applied Chemistry, Tohoku University, 6-6 Aoba, Aramaki, Aoba, Miyagi 980-8579, Japan; fukushima@aim.che.tohoku.ac.jp (J.F.); takizawa@aim.che.tohoku.ac.jp (H.T.); 2 Department of Chemical Science and Engineering, School of Materials and Chemical Technology, Tokyo Institute of Technology, Ōokayama 2-12-1, Meguro, Tokyo 152-8552, Japan; tsubaki.s.aa@m.titech.ac.jp (S.T.); matsuzawa.t.ac@m.titech.ac.jp (T.M.); fujii.s.ap@m.titech.ac.jp (S.F.); yuji-w@apc.titech.ac.jp (Y.W.); 3 Faculty of Engineering, Chubu University, 1200 Matsumoto-cho, Kasugai, Aichi 487-8501, Japan; 4 Research Institute for Sustainable Humanosphere, Kyoto University, Gokasho, Uji, Kyoto 611-0011, Japan; mitani@rish.kyoto-u.ac.jp (T.M.); shino@rish.kyoto-u.ac.jp (N.S.); 5 Department of Mechanical Engineering, Chubu University, 1200 Matsumoto-cho, Kasugai, Aichi 487-8501, Japan; t_namioka@isc.chubu.ac.jp; 6 National Institute of Technology, Okinawa College, 905 Henoko, Nago-city, Okinawa 903-0213, Japan

**Keywords:** microwave heating, electrical permittivity, graphite, shape effect

## Abstract

Microwave (MW) heating has received attention as a new heating source for various industrial processes. Some materials are expected to be a more effective absorber of MW, and graphite is observed as a possible candidate for high-temperature application. We investigated the dependence of the aspect ratio of graphite fibers on both their heating behavior and permittivity under a 2.45 GHz MW electric field. In these experiments, both loss tangent and MW heating behavior indicated that the MW absorption of conductive fibers increases with their aspect ratio. The MW absorption was found to be well accounted for by the application of a spheroidal model for a single fiber. The absorption of graphite fibers decreases with increasing aspect ratio when the long axis of the ellipsoid is perpendicular to the electric field, whereas it increases with the aspect ratio when the long axis is parallel to the electric field. The analytical model indicated that MW heating of the conductive fibers is expected to depend on both the shape and arrangement of the fibers in the electric field.

## 1. Introduction

Microwaves (MWs) have been extensively studied as an alternative energy source in applications such as chemical reduction and heating [1,2,3,4,5,6,7]. Wang et al. heated ceramic particles (ZnO, yttria-stabilized zirconia (YSZ), and Al_2_O_3_) by employing MW and conventional furnaces for sintering [4]. They reported low-temperature sintering of ZnO and large densification-enhancement of YSZ. Harutyunyan et al. reported a new scalable method for purification of single-wall carbon nanotubes by employing MW heating [5]. They insisted that MW treatment establishes a high local temperature on metal-catalyst particles; thus, they could remove the residual metal (Ni, Y) in arc-discharge carbon nanotubes to a level that is lower than 0.2 wt % (under 0.04 atom %). Yoshikawa et al. heated an NiO–graphite mixture, employed an electrical field separated from the MW, and reported enhancement in their reduction. They concluded that the phenomenon resulted from selective heating of graphite [6]. In the MW processing, reduction enhancement was observed in our system [7]. Many researchers have attempted to describe the mechanism of these MW enhancements.

The MW heating of a particle has been studied for several decades to understand their chemical behavior, which is an important property of MW heating [8,9,10]. Rybakov et al. [9] employed the Mie theory to describe the electromagnetic fields in cavities, where they disregarded the effects due to multipolar moments to simplify the solution. They obtained an analytical solution, which described the absorption by electrically conductive materials under different MW heating conditions. Experimental studies have also investigated MW heating mechanisms. Cheng et al. [10] compared the heating rates of various materials (metal, ceramics, and metal–ceramic composites) under different MW fields. By considering the energy loss of these materials in the MW fields, they found that the effect of the magnetic component cannot be ignored. Ma et al. [11] systematically investigated the absorption, heating behavior, and microstructure evolution of porous copper powder metal compacts subjected to 2.45 GHz MW radiation. They observed the heating behavior of copper particles with different radii. These reports indicated that MW heating of conductive particles is expected to depend on the shape of the particles and that the study of MW heating should progress to the next stage of determining the dependence on the microstructure and shape of these conductive particles.

In addition, the effect of relative densities and the shape of the powder on the MW absorption have been observed. To determine the effect of relative densities, Yoshikawa et al. measured the complex permittivity and direct current (DC) conductivity of FeO(OH)/C and trichloropropane/C mixtures, and the measured data were analyzed using percolation theory [12]. They analyzed the DC conductivity and permittivity using a mixing rule based on effective medium approximation. In this procedure, the permittivity of carbon was estimated by fitting the data of the measured average permittivity. The models reported by Levine and McQuarrie [13] are useful when the effects of the relative density on the absorption properties are considered. They expanded the Clausius–Mossotti function to account for the absorption behavior of a random distribution of metallic spheres. Recently, their model has been modified to estimate the dielectric constant of binary piezoelectric 0–3 composites by Jayasundere et al. [14], and the predictions by the modified Clausius–Mossotti function favorably agreed with the measured data of quasi-conductive particles [13].

In the present study, we experimentally investigated the MW heating and absorption characteristics of nonmagnetic conductive graphite fibers without natural oxide using pure graphite fibers as quasi-metallic fibers to observe their shape effects. We first investigated the effect of the shape on their absorption properties by comparing the results of their heating behavior obtained for graphite fiber compacts with various aspect ratios using spatially separated MW electric fields in an MW cavity. Next, their electrical permittivity was measured and compared with graphite fiber compacts with different aspect ratios using the cavity perturbation methods. The dependence of the aspect ratio on their absorption properties was considered by employing spheroid models.

## 2. Results

### 2.1. Theoretical Analysis

**Figure 1:**
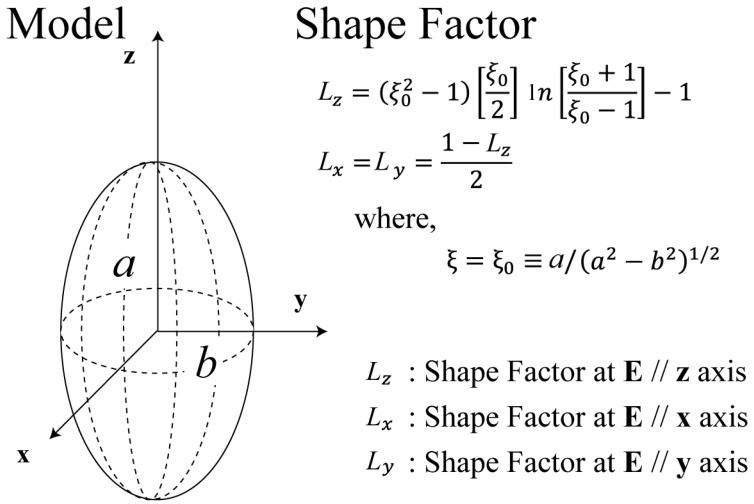
Spheroidal model and shape factors of the electromagnetic field absorption cross section. The shape factor indicates the orientation of the electric field vector along the long axis of the ellipse. The spheroidal model is described by Laplace’s equation as Mie theory, and the shape factors are obtained by employing variable transformation.

We briefly introduce the necessary theoretical background of the MW absorption by particles before discussing the MW absorption in graphite fibers. The shape effects of the particles on their absorption properties against electromagnetic fields are well known and are determined by the equation

(1) $$\alpha =\frac{V\left(\varepsilon_{1}-\varepsilon_{2}\right)}{L\left(\varepsilon_{1}-\varepsilon_{2}\right)+\varepsilon_{2}}$$

where *α* is the polarizability of the particle, *ε*_1_ is the permittivity of the particle medium, *ε*_2_ is the permittivity of the surrounding medium, *V* is the volume of a single particle, *L* is the shape factor of the particles, and the absorption cross section is an imaginary part of *α* (Im[*α*]). When a particle is spherical, the shape factor is one-third. Despite the difficulty in obtaining the shape factor using an analytical approach, our wavelength (MW frequency) is fortunately much larger than the particle size, and some analytical solutions were reported by employing quasi-electrostatic approximation [15]. We can employ a spheroidal model similar to the shape shown in Figure 1. The spheroidal model is described by Laplace’s equation as Mie theory, and the shape factors are obtained by employing a variable transformation. In this model, the shape factor indicates the orientation of the electric field vector with respect to the long axis of an ellipse [15,16].

(2) $$L_{z}=\left(\xi_{0}^{2}-1\right)\left[\frac{\xi_{0}}{2}\right]\ln\left[\left[\frac{\xi_{0}+1}{\xi_{0}-1}\right]-1\right]$$

and

(3) $$L_{x}=L_{y}=\frac{1-L_{z}}{2}$$

where *ξ*_0_ is the shape parameter of the ellipsoidal model, *a* is the length of the long axis of the ellipse, *b* is the length of the short axis of the ellipse, *L_z_* is the shape factor in the case where ***E*** // *z* axis, and *ξ*_0_ ≡ *a*/(*a*^2^ − *b*^2^)^1/2^. As *a* tends to infinity, these shape factors show the same behavior as that of the infinite-rod model. For fiber lengths that tend to infinity, these shape factors indicate the same behavior as that of an infinite-rod model. We employed the spheroidal model as an approximate approach, and the absorption properties of graphite fibers were investigated for all aspect ratios.

**Figure 2:**
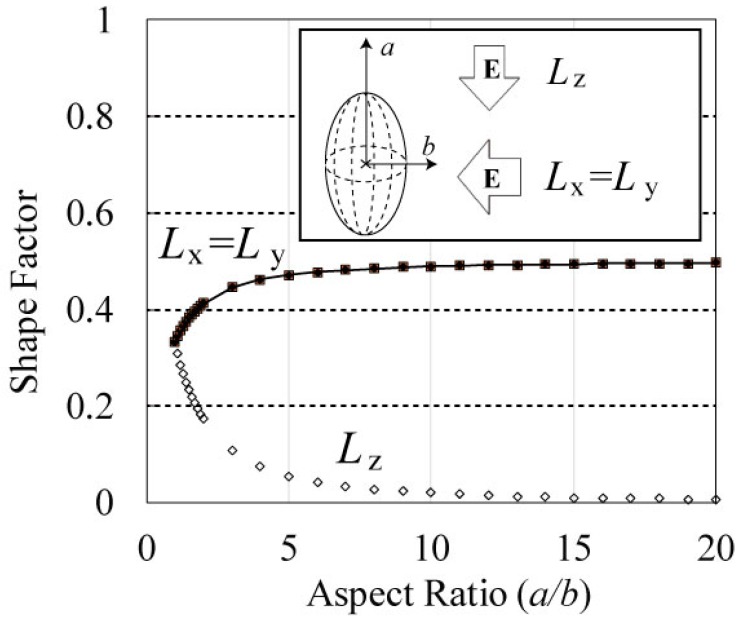
Shape factor of the spheroidal model versus aspect ratio. The shape factor, which determines the absorption properties of fibers, is changed by the direction of the ellipse relative to the electric field.

The discussion here will be focused on our system. Figure 2 shows that when the long axis of the ellipse is parallel to the electric field, the shape factor (*L_z_*) asymptotically decreases to zero with the increase in the aspect ratio. Because the absorption properties of the graphite fibers are linear with the inverse of their shape factors, as expressed in Equation (1), the decrease in *L_z_* (the denominator of the right term) indicates that its absorption cross section (=Im[*α*]) increases. Their absorption cross sections increase in this case. When the long axis of the ellipse is vertical to the electric field, the shape factors (*L_x_* = *L_y_*) asymptotically increase to one-half with increasing aspect ratio. In this case, the absorption cross section of the graphite fibers decreases as *L_x_* increases. Because the absorption cross section of the graphite fibers is defined by Equation (1), the decrease in *L_x_* (=*L_y_*) indicates that its absorption cross section (=Im[*α*]) decreases. Because *L_x_* (=*L_y_*) shows a constant value at an infinite aspect ratio, the cross section of the graphite fibers, which is vertical to the electric field, maintains absorption for MW in this case. The decreasing absorption cross section caused by the change in *L_x_* (or *L_y_*) indicates a constant value, whereas the increasing cross section caused by the change in *L_z_* becomes infinite corresponding to the aspect ratio. This result indicates that the average absorption of fibers (=1/3 Σ Im[*α*]) increases with aspect ratio. The absorption cross section of the particles, which is a loss factor of a particle, increases with the aspect ratio of the ellipse.

### 2.2. Experiments

**Figure 3:**
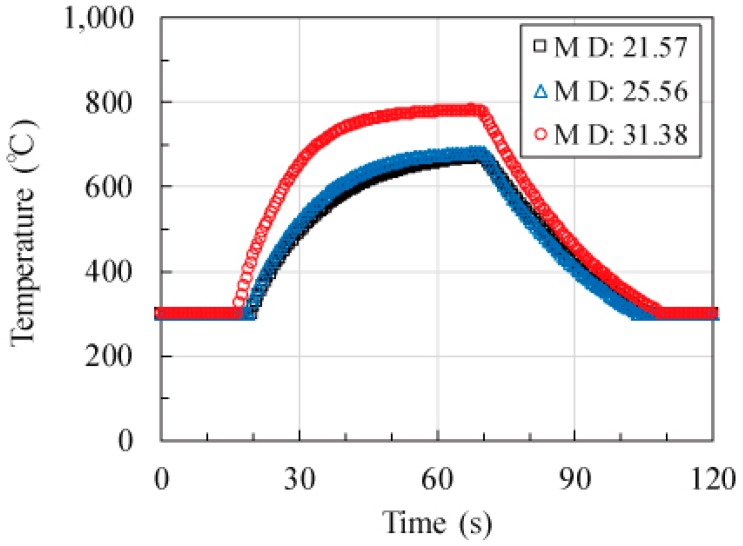
Time evolution of temperature for 15 vol % C fiber–Al_2_O_3_ mixture for MD = 21.57, 25.56, and 31.38 μm. The MW power is set to 40 W, and a 0.2 L·min^−1^ N_2_ gas is allowed to pass into the cavity.

The MW heating behavior conforms to the results obtained from the spheroidal model. Figure 3 shows the time dependence of the temperature for 15 vol % graphite fiber–Al_2_O_3_ mixture at mean volume diameter (MD) = 21.57, 25.56, and 31.38 µm (where MD is employed as the index of the aspect ratio because their MD is observed to increase with the aspect ratio in the scanning electron microscopy (SEM) observation, as presented in the Materials and Methods section). The results corresponding to these MD values are presented next. The maximum temperature is 670.8 °C, and the heating rate is 22.85 K·s^−1^ for MD = 21.57 µm. The maximum temperature is 681.5 °C, and the heating rate is 24.25 K·s^−1^ for MD = 25.56 µm. The maximum temperature is 782.0 °C, and the heating rate is 39.36 K·s^−1^ for MD = 31.38 µm. The MW heating behavior indicates that the maximum temperature and heating rate (300–400 °C) increases with MD. Because the maximum temperature indicates equilibrium between the input power to the fiber and the output power from the fiber and because the heating rates are inherently dependent on the absorption cross sections, the absorption cross sections of the fibers for MW radiation increase with their aspect ratios.

**Figure 4:**
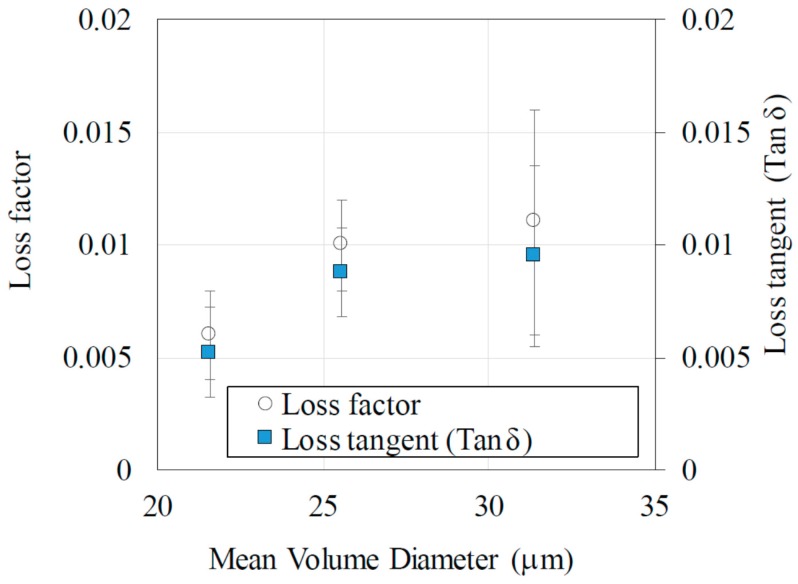
MD versus loss tangent (loss factor and their loss tangent) in graphite fibers (*N* = 7). The 30 mass % C fiber–SiO_2_ mixture is employed to observe the shape effects in the MW absorption properties. Both their permittivity and loss tangent (tan*δ*) increase with MD.

The relative permittivity was measured to investigate the MW absorption in graphite fibers because their absorption in the electric field is expressed in terms of the volume integration as 1/2*ωε*’tanδ *|E|*^2^, where tan*δ* is a function of *ε*″. The dependence of the relative permittivity and loss tangent on the MD are plotted and shown in Figure 4. Despite the theory that states that a small increase in the aspect ratio leads to a large improvement in the loss tangent (an MD increase from 21.57 to 25.26 makes the aspect ratio increase from 2.9 to 4.3 according to the SEM analysis, which results in a 30% increase in loss tangent), we still encounter difficulty observing the 30% increase in the loss tangent from the experiments. To eliminate this problem, we measured the loss tangent seven times in the experiment. For the 30 mass% graphite fiber–SiO_2_ mixture, the loss tangent (tan*δ*) doubles corresponding to the doubling of MD. We should note that the electrical conductivity of the compacts indicates insulation. The volume resistivities are 0.137, 0.049, and 0.012 Ω∙m for MDs of 21.57, 25.56, and 31.38 µm, respectively, for 100% graphite fibers, whereas they are the detection limits for the 30 mass % graphite fiber–SiO_2_ mixture. The result indicates that the effect of percolation conduction is not observed. Because MD is proportional to aspect ratio, the latter can be used to control the MW absorption using the electrical permittivity.

**Figure 5:**
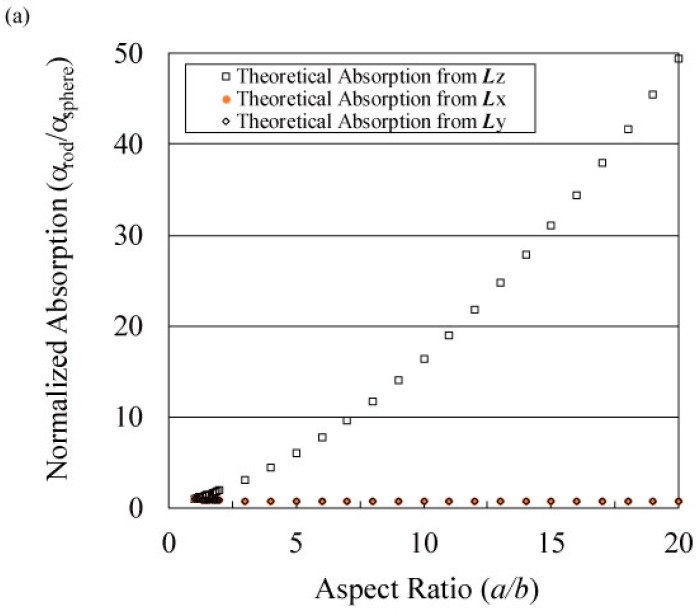

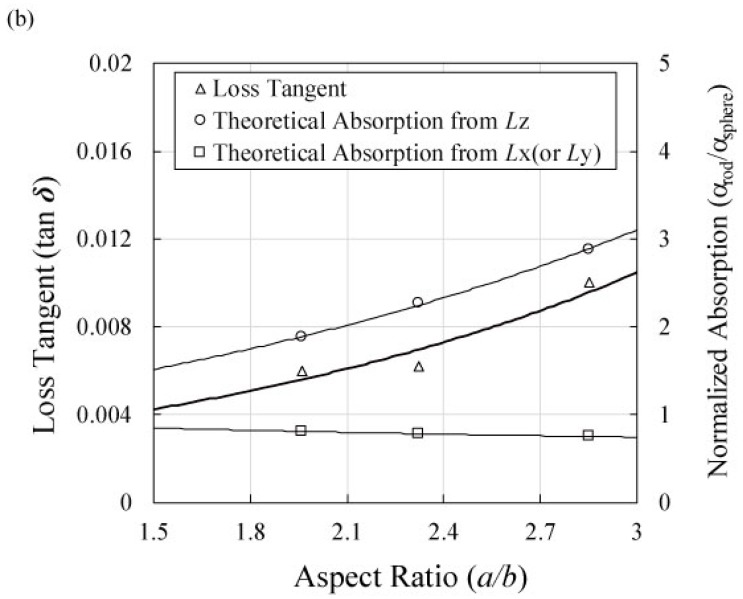
(**a**) Calculated normalized absorption from polarizability ratio (*α_rod_*/*α_sphere_*) versus aspect ratio (*a*/*b*) and (**b**) their comparison with the experimental data (tan*δ*).

Figure 5 shows the dependence of the calculated normalized absorption, expressed in terms of the polarizability ratio (*α_rod_*/*α_sphere_*), and the loss tangent on the aspect ratios (*a*/*b*) as well as their comparison with the experimental data (tan*δ*). *α* indicates the polarizability of the particles. In the following, *α_x_* indicates the normalized absorption from calculated *L_x_*. Normalized absorption *α_z_* exponentially increases with the aspect ratio, whereas *α_x_* and *α_y_* of the graphite fibers do not exhibit any variation. Figure 5b shows that the tan*δ* values are located between *α_x_* and *α_z_*. The simulated and measured tan*δ* values support our assumption that the absorption cross sections of the fibers for MW radiation increase with their aspect ratios because tan*δ* indicates the same tendency as that of the calculation, and both heating rates and maximum temperature conform to the measured tan*δ*.

**Figure 6:**
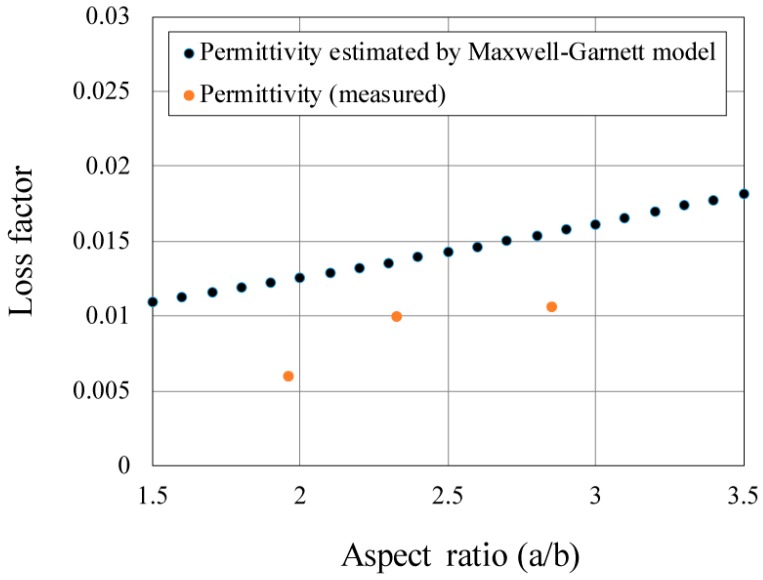
Dependence of the calculated loss factor obtained by the Maxwell–Garnett model and the measured values. The loss factor of the composite increases with increasing aspect ratio at low volume fractions of the graphite fiber, and the absorption of a single particle by a strong absorber largely affects their total absorption in a composite phase.

The Maxwell–Garnett model supports our proposal that the absorption of a single particle of a strong absorber largely affects their total absorption in a composite phase. Figure 6 shows the dependence of the calculated loss factor obtained by the Maxwell–Garnett model as well as the measured values. The Maxwell–Garnett expression for ellipsoidal fillers was described by Sihvola et al. [17] and is expressed as follows [18,19]:

(4) $$\varepsilon_{eff}=\varepsilon_{2}+\varepsilon_{2}\frac{\frac{\Theta }{3}\sum_{j=x,y,z}\frac{\varepsilon_{1}-\varepsilon_{2}}{\varepsilon_{2}+N_{j}(\varepsilon_{1}-\varepsilon_{2})}}{1-\frac{\Theta }{3}\sum_{j=x,y,z}\frac{N_{j}(\varepsilon_{1}-\varepsilon_{2})}{\varepsilon_{2}+N_{j}(\varepsilon_{1}-\varepsilon_{2})}}$$

where *ε_eff_* is the dielectric constant of the composite, Θ is the filler volume fraction (=0.15), and *N_j_* is the depolarization factor of ellipsoids in the *x*, *y*, and *z* directions. For needle-shaped fillers where radii *a* > *b* = *c*, *N_j_* has the following simple expression:

(5) $$N_{j}=(Ln((1+e)/1-e))-2e)(1-e^{2})/2e^{3}$$

where

(6) $$e=\sqrt{1-b^{2}/a^{2}}.$$

The permittivity of both graphite and quartz are 18 and 0.0063, respectively [20,21]. According to the results, the loss factor of a graphite fiber increases with increasing aspect ratio at low volume fractions, and the absorption of a single particle by a strong absorber largely affects their total absorption in the composite phase.

Our discussion will focus on the validity of the theoretical and experimental results. For the theoretical approach, the result of the spheroidal model agrees with the Maxwell–Garnett prediction. Considering that the heating behavior of the graphite fibers agrees with this theory, all results support our premise that the absorption cross section of the particles, which is the loss factor of a particle, increases with the increase in the aspect ratio of an ellipse. It can be noted here that the experimental results indicate lower absorption properties than the theoretical prediction shown in Figure 6 because the naturally filled fibers are slightly different in the isotropic arrangement.

The difference is believed to be due to the preferred orientation of the fibers. When we employed the Maxwell–Garnett prediction, the orientations of the fibers were assumed to be isotropic. From the spheroidal model, we could estimate how much the dielectric constant varied with the change in the fiber direction. When the aspect ratio (=*a*/*b*) was equal to 1.9, the model indicated that the loss tangent changed from 0.065 to 0.0195 (when the entire fiber was parallel to the electrical field, the loss tangent was 0.0195). At an aspect ratio of 2.8, the model indicated that the loss tangent changed from 0.075 to 0.0225, and the measured value existed in the region. However, in the experiments, the naturally filled fiber chose to have a low center of gravity and possibly became vertical relative to the electric field. Further, these fibers indicated lower absorption than the fibers that were parallel to the electric field. Because many fibers were vertical relative to the electric field in this state, the experimental results indicated lower absorption properties than the theoretical prediction.

By considering the effect of the shape of the fibers, we can possibly supply MW energy to specific particles in complex materials. When we consider MW heating for a good absorber at low density, the simple ellipsoidal model well describes the observed absorption properties, which result from the selective heating nature of MWs. The physical prediction becomes very useful in understanding industrial heating behavior. We believe that these results provide not only new data for carbon fibers, which are considered as an MW absorber, but also a simple model for predicting MW selective heating. We believe that we can heat a specific metal particle and a catalytic agent in complex materials and obtain a novel structure and a rapid synthesis by employing this effect. In fact, the absorption of graphite fibers is largely dependent on their aspect ratios, so their heating behavior can be controlled by controlling their aspect ratios.

## 3. Discussion

We determined the shape effects of nonmagnetic conductive fibers on the MW heating behavior. We investigated the dependence of the MD of graphite fibers on both their heating behavior and permittivity under a 2.45 GHz MW electric field. In graphite fibers, MW heats the graphite fibers of the MD because a twofold increase in the aspect ratios leads to a twofold increase in the loss tangent (tan*δ*). The application of the spheroidal model to a single fiber could account well for their absorption in these experiments. When the long axis of the ellipsoid is perpendicular to the electric field, the absorption of the graphite fibers decreases with increasing aspect ratio. Meanwhile, the absorption increases with the aspect ratio when its long axis is parallel to the electric field. However, when its long axis is parallel to the electric field, its absorption cross section increases with the aspect ratio. This model indicates that the loss tangent of the fiber sensitivity to MW increases with increasing MD. The MW heating of metal particles is expected to be affected by the shape of the particles, and the aspect ratio can control the MW absorption in terms of the electrical permittivity. In future research, MW heating should be studied in terms of other shapes and percolation effects.

**Figure 7:**
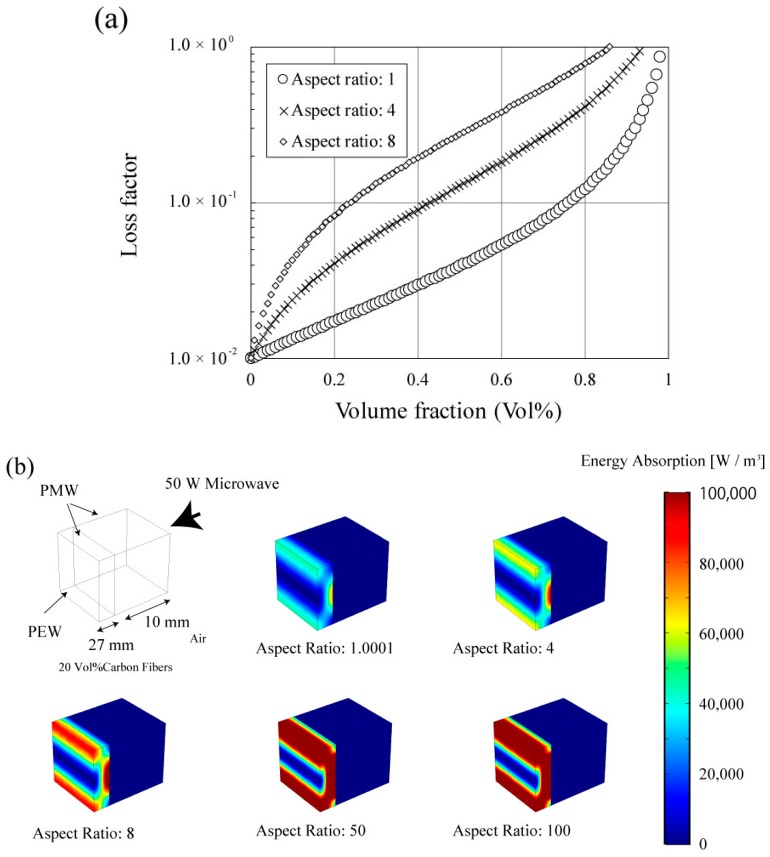
(**a**) Loss factor versus volume fraction calculated by the Maxwell–Garnet model and (**b**) electrical distribution in a large absorber with 20 vol % carbon fibers for each aspect ratio. According to the Maxwell–Garnet model, the equivalent dielectric constant of a 20 vol % carbon fiber greatly increases with the aspect ratio.

A λ/4 type radio MW absorber was employed as a simulation model. By controlling the shape of the carbon fiber, the MW absorption characteristic of the electromagnetic wave absorber can be improved as shown in Figure 7a. The Maxwell–Garnet model predicts that carbon fibers with an aspect ratio of 8.0 indicate a 10 times higher MW absorption capacity than carbon fibers with an aspect ratio of 1.0. When MWs are irradiated to a continuous body with this equivalent dielectric constant, their MW absorption increases the aspect ratio. For a 50 mm continuous body, the MW attenuation improves well in the calculation as shown in Figure 7b (where PEW denotes a perfect electrical wall, PMW denotes a perfect magnetic wall, and the simulation box size is 100 × 100 × 600 mm^3^. This λ/4 type radio MW absorber model was analyzed by the finite element method and COMSOL Multiphysics (COMSOL Multiphysics 5.3a, COMSOL Int.) was employed as a simulator). The absorption energy values for the model with aspect ratio 1.0001, 4, 8, 50, and 100 are 7.83, 10.91, 15.63, 34.23, and 36.330 W, respectively, and an absorption characteristic improvement of 5 times can be observed due to fiber length increases at the same carbon fiber volume density (where S11 values for the model with aspect ratio 1.0001, 4, 8, 50, and 100 are −0.73, −1.07, −1.63, −5.01, and −5.63 dB, respectively). In this calculation, as the aspect ratio increases, it is observed that the standing wave formed by the electric wall disappears. Although it is the same carbon fiber’s volume, it can be expected to increase the MW attenuation of the absorber by simply controlling the particle shape. Further, MW absorption can be expected by changing the containing particle shape.

## 4. Materials and Methods

**Figure 8:**
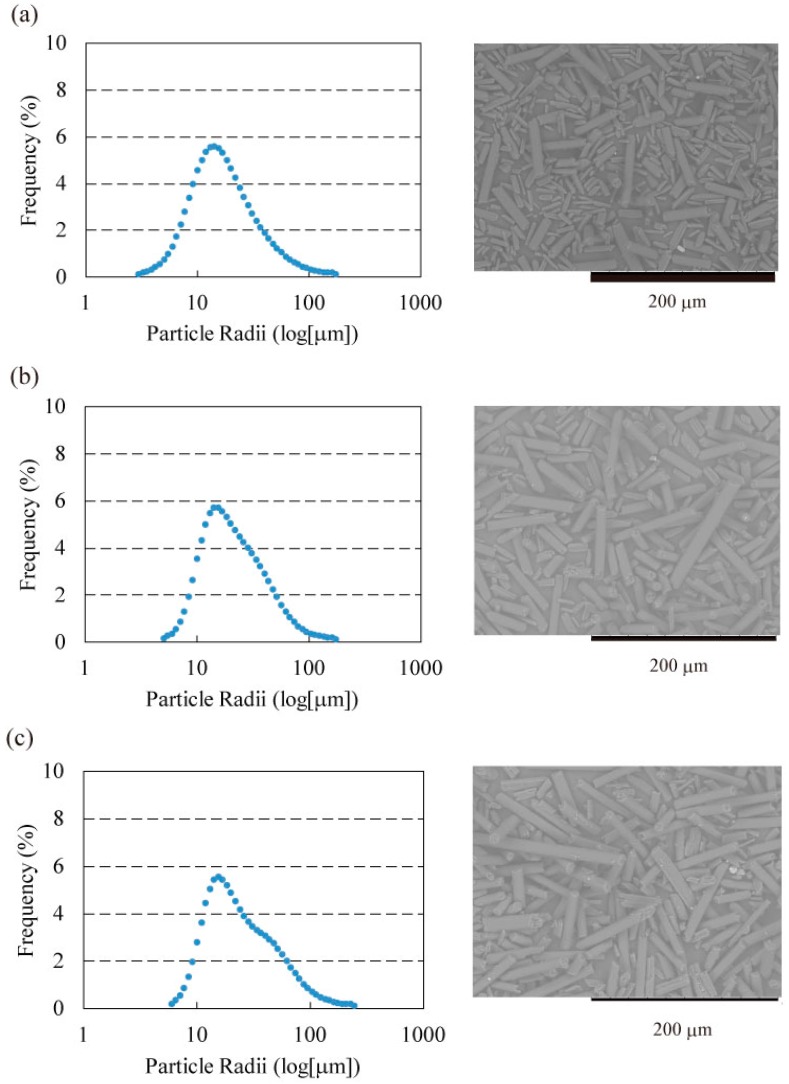
Particle size distributions of graphite fibers controlled by classification apparatus (Turbo Classifier, Nisshin Eng.) for (**a**) MD = 21.5 µm, (**b**) MD = 25.56 µm, and (**c**) MD = 31.38 µm. FESEM is employed to obtain the results of the light scattering for each treatment, as shown on the right-hand side of the figure. The fiber length decreases with increasing grinding time.

Graphite fibers with different aspect ratios were obtained by granularity control of K6371M fibers (Mitsubishi Rayon Co., Ltd., Tokyo, Japan; fiber diameter: 11 µm; average fiber length: 50 µm; resistivity: 6–7 µΩ·m; density: 2.1 g·cm^−3^; thermal conductivity: 140 W∙m^−1^∙K^−1^). The K6371 fibers were pulverized in a blender (WB-1, Osaka Chemical Co., Ltd., Osaka, Japan) for 1–4 min. Raw K6371 fibers, K6371 fibers pulverized for 1 min, and K6371 fibers pulverized for 4 min were classified based on their size using a centrifugal machine (Turbo-classifier 25, Nisshin Co., Ltd., Hyogo, Japan). The obtained fibers were then analyzed using both SEM (TM3000, Hitachi High-Technologies, Co., Ltd., Tokyo, Japan) and laser diffractometer (MT3300EXII, Microtrac Bell Co., Ltd., Osaka, Japan; solvent: H_2_O; refractive index: 1.33). Because their MD was observed to increase with the aspect ratio in the SEM observation, MD was employed as the index of the aspect ratio, and three fibers with good grain size distributions (MD: 21.57, 25.56, and 31.38 µm) were selected as sample fibers, as shown in Figure 8. We applied the microscopy method to the field emission SEM (FE-SEM) image to confirm that the aspect ratio increased with the MD. The averages of the aspect ratio were 2.9, 4.3, and 5.9 for MDs of 21.57, 25.56, and 31.38 µm, respectively.

**Figure 9:**
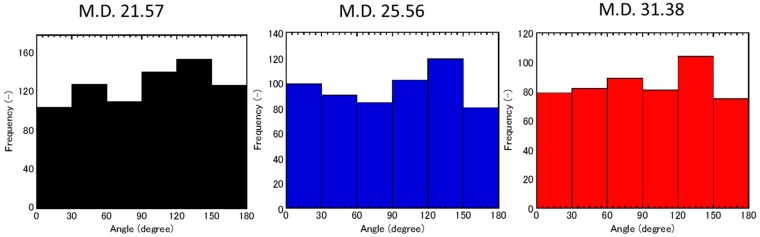
A histogram of orientation angle of the fibers in each pellet. A whole pellet was observed by SEM (×150) and angle was analyzed by using an image software. The orientation of fibers were in random state, which was assumed by Maxwell-Garnet model.

A fiber sample was heated using a single-mode applicator with 2.45 GHz MW irradiation from a magnetron. The heating system consisted of waveguides (109.1 × 56.4 × 149.3 ± 5 mm) with a magnetron oscillator, three stubs, a plunger, and an isolator. The MWs were focused by an iris and formed a TE103 wave in this cavity. The iris had a 28.5-mm-wide slit that was parallel to the direction of the electric field. The plunger was placed at the end of the waveguide. This system enabled us to spatially separate the electric and magnetic fields of the MWs [22,23]. The sample compact was a 15 vol % graphite fibers–Al_2_O_3_ powder (1 µm, Kojundo Chemical Co., Ltd., Saitama, Japan) to eliminate the effect of percolation because the percolation of electrical conductivities is often observed to have a volume ratio of over 30%. A 0.5 g sample was placed at an electric field node (denoted by *E*_max_, where the magnetic field is zero). As shown in Figure 9, the orientation of the fibers in the pellet was random at the plane, which is parallel to the surface (in this heating experiment, the fiber direction on the plane parallel to the pellet surface is important, because the vector of the microwave electric field is limited to one dimension parallel to the plane). The temperature of the reactants was monitored using a radiation thermometer with a lower limit of 200 °C (FTZ9-P300-20K21, Japan Sensor Corp., Osaka, Japan). Nitrogen gas was allowed to flow at a rate of 0.2 L·min^−1^ to prevent the graphite fibers from burning. The absorption MW power was fixed at 40 W (*P*_F_ = 183 W; *P*_R_ = 143 W) to generate high temperatures.

The real parts of the relative permittivity and loss factor of pure graphite fibers were measured to consider their heating mechanism. We used the cavity perturbation method [24,25] to obtain their values. The measurement system consisted of a square cavity and a network analyzer (ADVANTEST R3767CG, Keysigtt, Tokyo, Japan). Silica tubes have negligible effect on the MW distribution, so a silica tube was used as a sample holder. When 9 mm × 9 mm samples were moved into the cavity, they disturbed the *Q* factor and resonance frequency of the cavity because of their material properties. The network analyzer monitored the *Q* factor and resonance frequency of the system, and the real and loss factor of the relative permittivity (*ε_r_*′ and *ε_r_*″) were calculated based on these values. Because SiO_2_ powder is comparatively transparent to 2.45 GHz MW radiation, the sample compact was chosen to be a 10 vol % graphite fibers–SiO_2_ powder (<1 µm, Kojundo Chemical Co., Ltd., Saitama, Japan) to prevent measurement of the permittivity of the surrounding medium. The cavity exhibited a TM010 mode (in the TM mode, the Poynting vectors of the electromagnetic field were vertically controlled by the magnetic field), where a perturbation coefficient of 1.848 was employed. The conductivities of the carbon fibers were measured by a resistivity-measuring system (MCP-PD-51 equipped with Loresta GP, Mitsubishi Chemical Analytech Co., Ltd., Aichi, Japan).
